# Identification and Statistical Analysis of Impulse-Like Patterns of Carbon Monoxide Variation in Deep Underground Mines Associated with the Blasting Procedure

**DOI:** 10.3390/s19122757

**Published:** 2019-06-19

**Authors:** Justyna Hebda-Sobkowicz, Sebastian Gola, Radosław Zimroz, Agnieszka Wyłomańska

**Affiliations:** 1 Faculty of Geoengineering, Mining and Geology, Wroclaw University of Science and Technology, Na Grobli 15, 50-421 Wroclaw, Poland; justyna.hebda-sobkowicz@pwr.edu.pl (J.H.-S.); sebastian.gola@kghm.com (S.G.); 2 KGHM Polska Miedź S.A O/ZG, Polkowice-Sieroszowice, 59-101 Kaźmierzów, Poland; 3 Faculty of Pure and Applied Mathematics, Wroclaw University of Science and Technology, Wybrzeże Wyspiańskiego 27, 50-370 Wroclaw, Poland; agnieszka.wylomanska@pwr.edu.pl

**Keywords:** gas hazards, carbon monoxide, mine, impulsive behaviour, process identification, segmentation

## Abstract

The quality of the air in underground mines is a challenging issue due to many factors, such as technological processes related to the work of miners (blasting, air conditioning, and ventilation), gas release by the rock mass and geometry of mine corridors. However, to allow miners to start their work, it is crucial to determine the quality of the air. One of the most critical parameters of the air quality is the carbon monoxide (CO) concentration. Thus, in this paper, we analyze the time series describing CO concentration. Firstly, the signal segmentation is proposed, then segmented data (daily patterns) is visualized and statistically analyzed. The method for blasting moment localization, with no prior knowledge, has been presented. It has been found that daily patterns differ and CO concentration values reach a safe level at a different time after blasting. The waiting time to achieve the safe level after blasting moment (with a certain probability) has been calculated based on the historical data. The knowledge about the nature of the CO variability and sources of a high CO concentration can be helpful in creating forecasting models, as well as while planning mining activities.

## 1. Introduction

In underground mining, the gas hazard, i.e., the presence of concentrations of harmful substances such as carbon monoxide, methane or hydrogen sulphide often might influence mining production. All these harmful gases are important in order to identify the safety of working space. Each of them in high concentrations can cause health problems or even death. Moreover, CO is a colorless, odorless and tasteless compound often referred to as the “*silent killer*” because it is undetectable by people without using a detection device. Exposure to high concentrations, which are 100–300 ppm (part per million), for 2–3 h may cause headaches. What is worse, a prolonged exposure to unsafe carbon monoxide levels can cause permanent health debility, such as memory impairment, mental disability, loss of appetite, loss of sensation in the fingers, drowsiness during the day and insomnia at night, as well as circulatory disorders. In this paper, we focus only on the CO concentration in order to find the complete description of its variation in the considered underground mine (see Section 2.1), where it is known as a major gas hazard. The analysis of the other gases will be the material for future papers.

To overcome the gas hazards, some actions have to be done, namely providing the appropriate fresh air volume to operation fronts (mining faces) and determining the proper waiting time after blasting operations to effectively ensure the safety of miners. An experience of miners in recognition and mitigation of gas hazard shows that the factors influencing the occurrence of gas concentrations are: Type and tectonics of roof rocks, changes in the atmospheric pressure, geometry of the mining technology (excavation systems) used in the mine, the direction of the mining front in relation to the geometry (extent) of the deposit and the rock cracks rose, the method of the ventilation used in the mine, the inclination of the exploitation (mining) front, the way of liquidation of the empty space, the speed of progress of exploitation fronts and elimination of the empty space. The mixed influence (with unknown, time-varying coefficients) of these factors causes a high variability of gas concentration and varying duration of the gas presence. In order to summarize such a complicated challenge, it is worth highlighting the following critical issues.

Firstly, blasting events produce a lot of gases, and there is a need to use ventilation techniques to return safe conditions. It is critical, due to the fact that the mine is deep, that the total length of the ventilation channel (from the mining face to the ventilation shaft) is long and therefore the ventilation process is time-consuming and extremely expensive. Miners are not allowed to start their work until the parameters of the mine’s atmosphere are within acceptable limits. Obviously, it is nonproductive time. According to our knowledge, there is no systematic research (based on the long-term measurement and statistical analysis) in this field, in particular for CO concentration. However, similar papers related to the gas hazards we can find in [1,2,3,4].

Secondly, even if a high gas concentration, produced by the blasting, will decay after some time, there are other fluctuations, that sometimes may exceed the safe level of CO concentration. Unfortunately, due to the geometry of the mine, networks of corridors, total lengths of tunnels, nowadays, it seems to be impossible to have a spatial distribution of such parameters in real time. The most critical part of the mine, i.e., the vicinity of mining faces, has no infrastructure (sensors, monitoring systems, electricity, etc.), so the only possible solution is to have individual gas detectors for each miner. It might partially help; however, it doesn’t provide historical data for deeper, more comprehensive analysis. Thus, we want to model and analyze it statistically, based on historical data to get the basic knowledge about its behaviour. To face such a challenge, an experiment is performed in the mine (described in Section 2).

In this paper, we will investigate the first (probably more important due to the dynamics of the process) part of daily variation related to impulse-like behaviour, caused by the blasting event. In order to understand the properties of such processes, we will start with detection and segmentation methods. There are many great examples of advanced analytics for industrial/environmental/mining data that cover classical problems for streaming data such as validation, cleaning, segmentation, de-noising, filtering, detection and approximation [5,6,7,8,9]. In this paper we base on known and tested methods, modified for the need for thorough analysis. The appropriately pre-processed signal could be further analyzed using time series or statistical models techniques [10,11,12,13,14,15,16,17,18] to answer how big the peak can be, how fast it will decay and finally—when the safe value (26 ppm, according to the regulation of the Minister of Energy related to operations of underground mining [19]) of CO concentration will be achieved after blasting. Such knowledge is very important for daily decisions [19], as well as, for long-term strategic mine planning. Understanding the nature of the CO variation and sources of the high CO concentration might be also helpful to build prognostic models [20,21].

The rest of the paper is organized as follows. In Section 2 we describe the experiment. Section 3 presents the methodology used for the data analysis. In the next section we present the real CO data analysis and give the first conclusions. The last section is a summary with underlined practical information, extracted from data analysis.

## 2. Experiment Description

In the deep underground mine considered in this paper, a room and pillar mining technology with blasting is used. Moreover, LHD machines (loaders, trucks) with diesel engines are operated for transport of ore from mining face to conveyor chains. The most important sources of gas hazard here are related to the technological processes, mainly gases issued from explosive materials and exhaust gases, from LHD engines. Referring to the previously listed gas hazards, we want to highlight the importance of CO concentration in the considered underground copper ore mine. The quantities of carbon dioxide produced during mining work has limited influence to air quality, moreover CO${}_{2}$ is not poisonous gas. Significant increase of CO2 potentially might lead to change of the percentage of air content (less than 19% of oxygen in the atmosphere is dangerous); however, in the considered mine it has never appeared. The hydrogen sulphide concentrations are the result of phenomena occurring in the rock mass and based on measurements of the concentrations of this gas one can conclude about the level of mining crew hazards [4]. However, the hydrogen sulphide in small concentrations can alert about its presence by releasing a very specific smell of rotten eggs. Last but not least is methane, but it has not occurred in the considered copper ore mine so far. As for other gases, their concentrations are below the thresholds of quantification. As one could see, the CO as the "*silent killer*" seems to be the most dangerous.

An experiment has been carried out in one of the mining units in a deep underground copper ore mine during the period 28 October 2014–28 December 2014. It monitors several physical parameters in order to investigate how these parameters are changing over time. According to our knowledge, the installation of the measurement device with several gas-type detectors and long-term data acquisition is the first instance in the history of copper mining in Poland. Details of the experiment will be presented later in the paper. Practically, before miners get access to the mining face, the mining engineer and the supervising the team of miners have to measure air quality by portable equipment and give the permission for the rest of the team. It is inconvenient and repeats once per shift. In the considered underground copper ore mine the time equal to 30 min was the most common waiting time after blasting to allow miners entry into the place. This usually-accepted waiting time was based on historical measurements, which have been performed by the engineers several times during the day before allowing miners to start their work. What is more, the level of gas may change during the shift. Thus, there is a need to know the general behaviors of the gas variation. However, in order to protect miners during a sudden increase of the CO concentration, they are equipped with individual equipment, such as half masks with filter-absorbers and gas-tight safety goggles.

This section will discuss three main issues related to the experimental part; namely, we will present in detail the geometry and the conditions in the mining unit in the considered mine; next, briefly, the measurement system will be discussed, its location will be pointed out; and, finally, some basic parameters of the monitoring device will be presented.

### 2.1. Mining Area for Experiment

**Figure 1:**
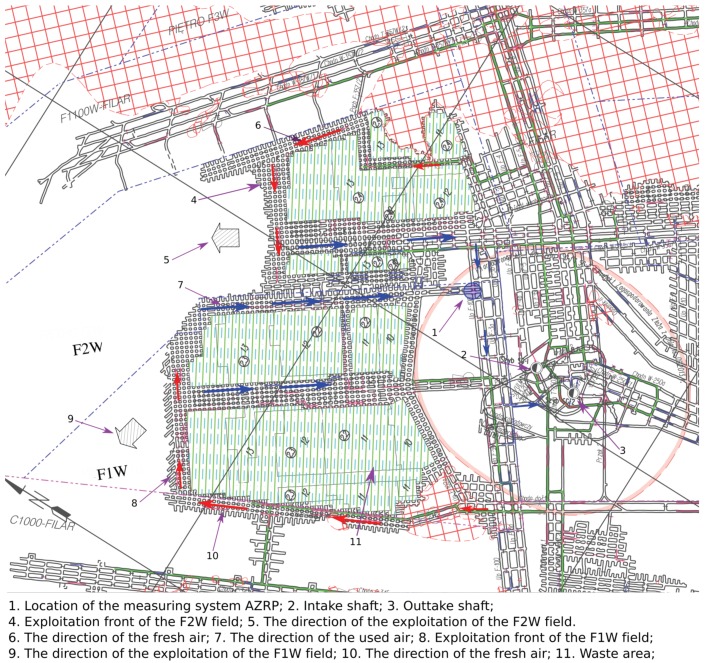
Map of the investigated part of the mine with fresh and used air stream flow directions and sensors location.

The considered mine is one of the most difficult due to its size, depth (and perspective to be deeper) and harsh environmental conditions (high temperature of rock mass and of air in mining voids, harmful gases and other natural hazards). As mentioned, in the analyzed mine, room and pillar mining technology with blasting is used. The explosives are an unavoidable part of extracting the copper ore. The parameters of the explosive material are precisely described by the mining regulations and in each case of the performed explosion, the type of material is the same and is provided by the same company, so they should not affect the variability of the process. After mining, the back-filling technology is used (waste rock is used, see depicted green areas in Figure 1). As presented in the map, see Figure 1, in the considered area there are two active mining fronts, used and fresh air streams, depicted with blue and red arrows, respectively. The blue circle, pointed by the arrow numbered as 1, is the location of sensors. They are installed in one of the chambers in the mining field F1W. Due to mining technology and a number of possible ways for air streams, the location of the sensors should be considered as a compromise.

In the analyzed period, an air flow of amount 4800 m${}^{3}$/min flowed through the operating field. The air to the unit is supplied from the SG-1 intake shaft (indicated in Figure 1 with the arrow number 2 to the exploitation front of the F2W field (arrow number 4) and the exploitation front of the F1W field (arrow number 8) with excavations with a total length of approximately 5 km. The bed is cut into pillars with dimensions of 8×7 m, (length × width—length is the side of the pillar located parallel to the direction of the exploitation front), with the location of the long axis perpendicular to the line of the designed belts. The width of chambers and belts is 5 m (using this information we can estimate the size of places included on the map (Figure 1). The additional information about this mine is as follows:

- The deposit depth is 1026 ÷ 1081 m
- The extension of the deposit in general is NW-SE, the fall 2÷6° on NE, the slope locally exceeds the inclination of 8°
- The daily extraction of 4200 Mg.

### 2.2. Measurement System

**Figure 2:**
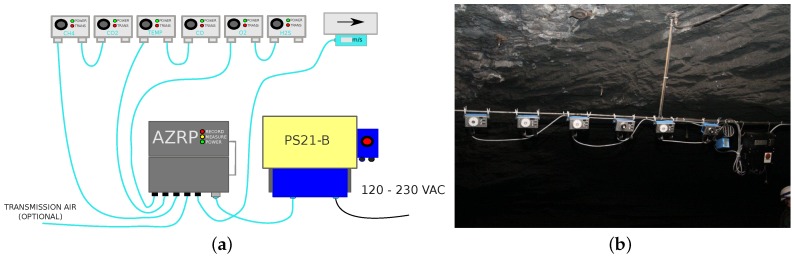
Measurement system used in the experiment. (**a**) Measurement system—scheme [25], (**b**) photo of sensors installed in the mine.

Thanks to the rapid development of sensors technology, physical phenomena that occur in the real world could be measured nowadays. We can find great examples where a network of sensors with their use are discussed. While advanced data acquisition systems are available, extremely harsh conditions in the mine (especially deep mine) and the complex nature of processes bring a lot of challenges [22,23,24]. The monitoring system used in this experiment is able to measure CO, CO${}_{2}$, H${}_{2}$S, CH${}_{4}$ and other physical variables such as humidity, temperature, and air stream speed. The purpose of this paper is to use the collected CO concentration data. The vector of observations contains the concentration of the carbon monoxide in deep underground copper ore mines from the given period 28 October 2014–28 December 2014. The frequency of data acquisition is 1 s. In this specific place, there is no electric infrastructure, thus once a month a measurement is stopped and data are transferred from the device to the computer by an SD card. Structure of the monitoring system is presented in Figure 2a and the corresponding sensors’ installation in the considered mine is presented in Figure 2b.

It is obvious that the system has to be robust to manage the harsh conditions existing in the mine. The device is manufactured by the Sevitel company. It uses electrochemical sensors. Sevitel conducts activities in the field of design, production, prefabrication, installation and service of innovative solutions in the area of monitoring, automation and control, allowing to increase safety in industrial plants and public facilities. The intrinsically safe carbon monoxide concentration sensor is a device designed to measure the concentration of carbon monoxide in the atmosphere in the range of 0–1000 ppm in industrial facilities with specific methane and/or coal dust explosion hazards, as well as specific environmental conditions, e.g., high ambient temperature, high humidity and high dustiness.

The device is made in an intrinsically safe standard and has an extended temperature range (−20 °C÷+50 °C) and an increased degree of protection against penetration of external factors (IP65) [19] (IP, i.e., Index of Protection). The measurement device was developed by Sevitel for mining applications and takes into account specific mining demands, such as high selectivity—in case of presence of several gases they are able to properly measure all of them, short response time, the ability to read measurement results from all sensors in a short time (minimum 1 s), low energy consumption, small dimensions and weight. The information about the current operational status of devices is signaled by LED diodes. The most important parameters of the sensor have been listed in Table 1.

The sensor has all the necessary certificates and meets the standard T90 [26]. Additionally, in order to obtain correct readings of measured gas concentrations throughout the entire measurement period, the sensors were disassembled once a month to be checked and calibrated directly by the manufacturer.

## 3. Methodology

**Figure 3:**
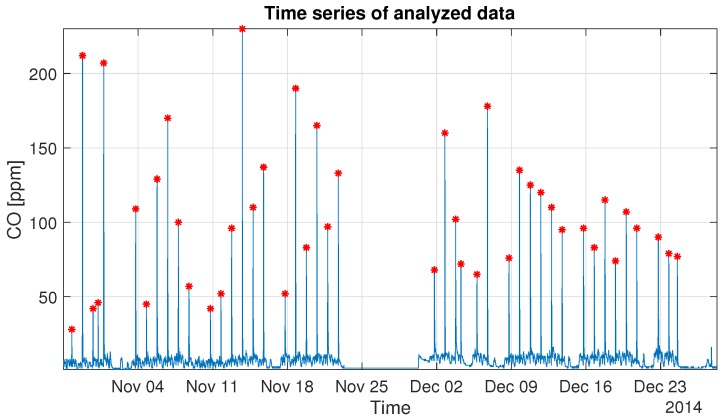
Raw signal describing carbon monoxide variation.

**Figure 4:**
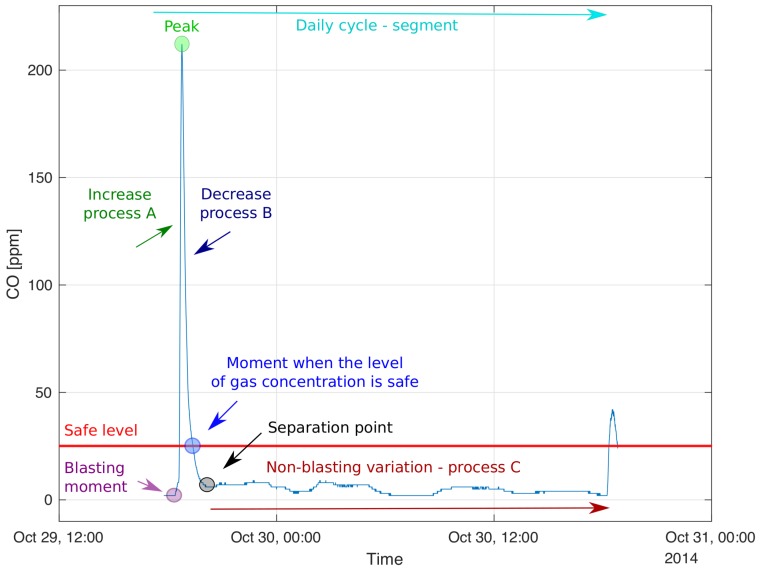
The schematic idea of the analyzed process responsible for the carbon monoxide concentration.

In this section, we present the methodology used in real data analysis. The goal of this analysis is to properly describe the selected parts of the process responsible for carbon monoxide variation. In the raw CO data, see Figure 3, there are clearly visible parts which cannot be described by the same process. Our vision is to perform the segmentation of the vector of observations and to propose a mathematical description of each individual segment. As one can see in the time plot of the analyzed real-data, see Figure 3, there are visible very high local gas concentrations, which are related to the blasting. In the examined vector of observations, we can detect 44 similar peaks. Each of them has a complicated structure, but repetitive. Namely, the process visible just before the peak has a different nature than the process just after it. Moreover, the process between the peaks is also different because it is induced by different sources than the processes mentioned earlier. However, it is also included in the processes around the peak but not visible because of the overwhelming energy of the peak relative to this process of a plateau. The occurrence of small concentrations of carbon monoxide, which change according to the visible plateau, can appear due to operating mining machines, as well as from the slight release of this gas from the rock mass. Considering the above, in order to describe the nature of those processes, there is a need to apply different mathematical methods. The schematic Figure 4 shows the idea.

In this paper, we focus on the analysis of the processes just after peak, namely process B, which is responsible for the decreasing of the carbon monoxide concentration. This is a process visible in the period between the maximum value of the peak and the point where the carbon monoxide concentration reaches the separation point, namely the place where we observe the inflexion of the extinguishing function and a new slight increase in the CO concentration is visible. Probably, it is the place where the natural variation of the carbon monoxide level is the predominant process. Moreover, we will examine the probability distribution of the peaks amplitude and calculate how long miners should wait to safely enter the workplace after blasting. In order to proceed these steps we also aim to define the automatic procedure for indicating the blasting moment and separation point. This analysis will help to understand the nature of the CO variation and predict its values. The processes A and C are not examined in this paper. The corresponding analysis will be presented in our future research.

In the first step, we detect the large observations in the analyzed time series. In order to do this, we apply local maximum method, allowing us to find the place where the value of the carbon monoxide is the largest for the given time interval. The necessary assumptions we describe in Section 4. In order to validate peaks’ locations detection, data from the seismic monitoring system used in the mine (each blasting is noticed by the seismic system) are investigated and compared. In general the amplitude of peaks might depend on the amount of the blasting material, distance between mining face, sensor location and geometry of the mine corridors (that are slightly varying). Thus, first we examine the distribution of the amplitudes of the peaks. Here we apply the standard method based on the observation of the empirical tail. More precisely, if $a_{1},a_{2},\cdots ,a_{n}$ are the detected peaks amplitudes, then we calculate their empirical tail, assuming they constitute a sample of independent observations. The empirical tail is defined as follows:

(1) $$\begin{array}{c} ET\left(x\right)=1-{\hat{F}}_{n}\left(x\right)=1-\frac{1}{n}\sum_{i=1}^{n}1_{\left\{a_{i}\leq x\right\}}, \end{array}$$

where *x* is the argument of the empirical tail function and $1_{\left\{A\right\}}$ is the indicator of the set *A*. In (1) the ${\hat{F}}_{n}\left(\cdot \right)$ is the empirical cumulative distribution function for sample $a_{1},a_{2},\cdots ,a_{n}$. In order to recognize the class of distribution corresponding to the vector $a_{1},a_{2},\cdots ,a_{n}$, we fit the theoretical tails of known distributions to the empirical tail. In our case we take under consideration the following non-negative distributions, defined through cumulative distribution functions F(·) as follows [27]:

1. Log-normal:
  (2) $$\begin{array}{c} F\left(x\right)=\frac{1}{2}+\frac{1}{2}\mathrm{erf}\left[\frac{\ln(x)-\mu }{\sigma \sqrt{2}}\right],\;\mathrm{for}\;\left\{\sigma ,x\right\}>0\;\mathrm{and}\;\mu \geq 0. \end{array}$$
2. Burr:
  (3) $$\begin{array}{c} F\left(x;c,k,\alpha \right)=1-{\left[1+{\left(\frac{x}{\alpha }\right)}^{c}\right]}^{-k},\;\mathrm{for}\;\left\{c,k,\alpha \;x\right\}>0. \end{array}$$
3. Pareto:
  (4) $$\begin{array}{c} F\left(x,x_{m},\alpha \right)=1-{\left(\frac{x_{m}}{x}\right)}^{\alpha },\;\mathrm{for}\;x\geq x_{m}\;\mathrm{and}\;\left\{\alpha ,x_{m}\right\}>0. \end{array}$$
4. Gamma:
  (5) $$\begin{array}{c} F\left(x,\alpha ,\beta \right)=\frac{1}{\Gamma (\alpha )}\gamma \left(\alpha ,\beta x\right),\;\mathrm{for}\;\left\{\alpha ,\beta ,x\right\}>0. \end{array}$$
5. Weibull:
  (6) $$\begin{array}{c} F\left(x,\lambda ,k\right)=1-e^{-{\left(x/\lambda \right)}^{k}},\;\mathrm{for}\;\left\{\lambda ,k,x\right\}>0. \end{array}$$

We mention the theoretical tail of given distribution is just 1− its cumulative distribution function.

In the analyzed case we confirm our visual test of distribution based on the comparison of the empirical and theoretical tails by the classical goodness-of-fit test, namely Kolmogorov-Smirnov test [28].

For the vector of the independent observations $a_{1},\cdots ,a_{n}$ the most used version of the Kolmogorov-Smirnov test compares vector of independent observations of empirical cumulative distribution $\hat{F}\left(x\right)$ function against a known Gaussian cumulative distribution function Φ(x). Thus the Kolmogorov-Smirnov statistic is:

(7) $$\begin{array}{c} KS=\underset{x}{\sup}\left|{\hat{F}}_{n}\left(x\right)-\Phi \left(x\right)\right|. \end{array}$$

The test statistic from Equation (7) is a base to calculate the *p*-value. For the Gaussian cumulative distribution function Φ(x) the distribution of this Kolmogorov-Smirnov (KS) statistic is commonly known, see [28]. Therefore one can calculate the corresponding *p*-values by using explicit formulas.

In the classical version, the Kolmogorov-Smirnov test is applied to the problem of Gaussianity testing. However, one can extend it to any other probability distribution. The corresponding *p*-value can be calculated using Monte Carlo simulations. This is the case considered in this paper. We remind that the general goodness-of-fit test is structured as follows. The null hypothesis $\left(H_{0}\right)$ is that a specific distribution is acceptable, whereas the alternative $\left(H_{1}\right)$ is that it is not. Small values of its test statistic, in our case KS, are evidence in favor of the hypothesis, a large one indicates its falsity. In other words, the small value of the KS indicates that the empirical and the theoretical (fitted) distributions are closed. This leads to the large *p*-value, thus the $H_{0}$ hypothesis is not rejected. To see how unlikely such a large outcome would be if the hypothesis is true, we calculate the *p*-value by:

(8) $$\begin{array}{c} P(D\geq d), \end{array}$$

where *D* is the test statistic and *d* is the statistic value for a given sample.

It is typical to reject the hypothesis when a small *p*-value is obtained, like, for example, below 3% or 5%. In our analysis, we used the significance level equal to 5%.

After recognition of the probability distribution for peaks’ amplitudes, we analyze the process after peaks. In Section 4 we give the description of how to find the point which starts the process before and ends the process after the peak. In the examined real-data we observe process B (responsible for decreasing to the separation point), which is mostly deterministic. Therefore we propose to describe it by the deterministic functions. We apply the least squares method to estimate the corresponding parameters. The examined function we present in Section 4. However, during the analysis of the time series of the process after peaks, we recognized they can be described by using the same model but with different parameters. In order to prove this assumption, we applied the Silhouette algorithm [29], which returns a number of classes corresponding to the analyzed data. If the algorithm returns one class, then we assume the data are homogeneous. For a fixed number of classes *c* the Silhouette statistic assigns value $s_{c}\left(i\right)$ to the observation $\phi_{1},\cdots ,\phi_{m}$, given by:

(9) $$s_{c}\left(i\right)=\frac{d_{1}\left(\phi_{i}\right)-d_{2}\left(\phi_{i}\right)}{\max\{d_{1}\left(\phi_{i}\right),d_{2}\left(\phi_{i}\right)\}},\;\;i=1,2,\ldots ,m,$$

where $d_{1}\left(\phi_{i}\right)$ is the average distance to all values in the allocated class and $d_{2}\left(\phi_{i}\right)$ is the distance to the nearest neighbor class. For each number of classes *c*, all possible divisions into *c* classes are considered and the optimal division is the one that maximizes the Silhouette statistic $s_{c}$. The Silhouette criterion then takes a value:

(10) $${\mathrm{sil}}_{c}=\sum_{i=1}^{m}s_{c}\left(i\right)/m,$$

that varies from 0 to 1. The optimal number of classes maximizes ${\mathrm{sil}}_{c}$.

**Figure 5:**
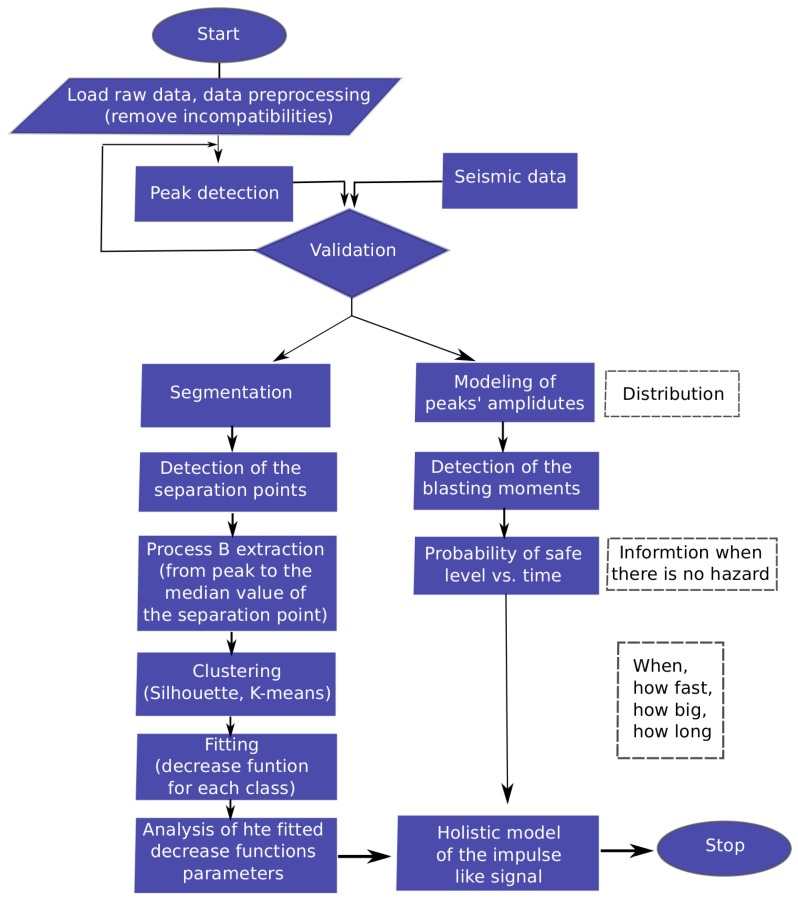
The block diagram of real data analysis.

After confirmation that the processes responsible for the decreasing carbon monoxide concentration belong to different classes, we classify them using K-means algorithm [30]. In K-means algorithm the assignment into a given class is based on the minimization of the criterion that within each cluster calculates the average Euclidean distance between the points in that cluster and observations from the cluster mean. In the real-data analysis, we confirmed the two classes’ existence, describing the processes after peak by fitted deterministic functions, which coefficients take different values for different classes. The schematic algorithm of the real data analysis we present in Figure 5.

## 4. Real Data Analysis

In this section, we present the real-data analysis, using the methodology described in the previous section. In Figure 3 one can see the time-plot of the examined data of the carbon monoxide concentration. It is easy to notice that the time series contains characteristic jumps, associated with explosions derived from technological processes. Each of the explosion, caused by an explosive charge, generates the emission of many chemical compounds, including carbon monoxide, which significantly increases its concentration in the air.

The vector of observations contained missing values, so a pre-processing was needed. For cases where the gaps have a small time interval, we fill the missing observations using interpolation based on adjacent values, otherwise, we do not take such periods into account. As one can observe in Figure 3, the longest data gap appears from 23 November 2014, 07:31:30 to 30 November 2014, 07:06:04. It is the period when the measurement system had been disassembled for a calibration and for data collection. In the further analysis, we do not take into account these missing days. After pre-processing, we started the analysis from the location of the visible peaks and their values in order to describe the probability distribution of these amplitudes and to validate if all of them come from the blasting moments. To find the location and values of peaks the method of a local maximum was used. In the procedure of detection of peaks locations we take the following assumptions:

1. Peaks’ amplitudes are greater than 26 ppm. This is a widely accepted limit value of the carbon monoxide concentration allowing miners to stay in such air conditions.
2. Peaks do not appear more often than every 7 h (selected arbitrarily—this number cannot be too large so as not to omit the significant peaks, but also it cannot be too small, then the local maximum method could take the maximum as the end of the moving window, if there is no peak inside).

**Figure 6:**
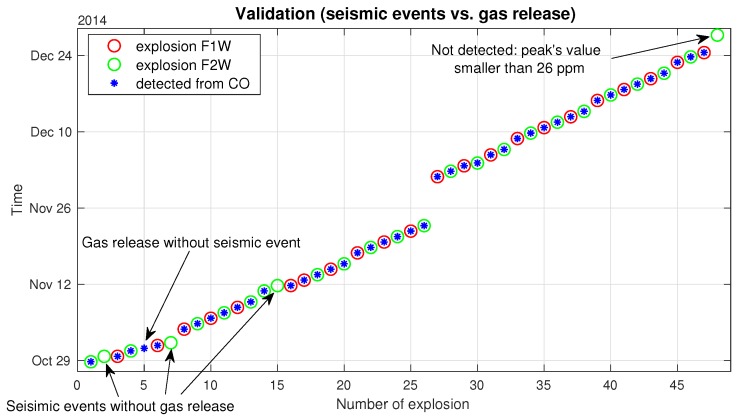
Peaks’ detection validation: Comparison of the detected peaks’ locations with the data from the seismic monitoring system.

The number of detected peaks, presented in Figure 3 (red stars), is equal to 44. In order to validate peaks’ locations detection, the results have been compared with data from a seismic monitoring system used in the mine (each blasting is noticed by the seismic system). As one can see in Figure 6, most of the detected peaks coincide with the blasting shocks; however, one of the carbon monoxide emissions was not registered by the seismic monitoring system (5th peak, marked as the blue star), which we understand as the natural gas emission from a rock mass. On the other hand, the four seismic vibrations occurred without carbon monoxide volatilizing (all of them come from the F2W field, labeled in Figure 1), the first three we combine with typical intrinsic seismic vibration. The last one was not detected in CO time series data because it did not exceed the safe level of the carbon monoxide concentration.

The graphical test, as well as the KS test, have been used as the indicator for the best fitting function selection among 5 considered (described in Section 3). Based on the graphical results, it is easy to observe that Pareto distribution fails. The goodness of fitting of the other distributions is similar. The Burr distribution has been chosen as the best-fitted one. To improve the goodness of fitting, the KS test (described in Section 3) based on the empirical data and data simulated from Burr distribution with the estimated parameters has been performed. The *p*-value of the test was calculated by using 1000 Monte Carlo simulations. The median of the obtained *p*-values is equal 0.82 which indicates the $H_{0}$ hypothesis is not rejected at given confidence level 5%. The values of fitted Burr distribution parameters are as follow:

- Scale parameter α=284.77.
- Shape parameters c=2.42 and k=8.49.

They correspond to the cumulative distribution function $F\left(x;c,k,\alpha \right)=1-{\left[1+{\left(\frac{x}{\alpha }\right)}^{c}\right]}^{-k}$ (described in Equation (3)).

**Figure 7:**
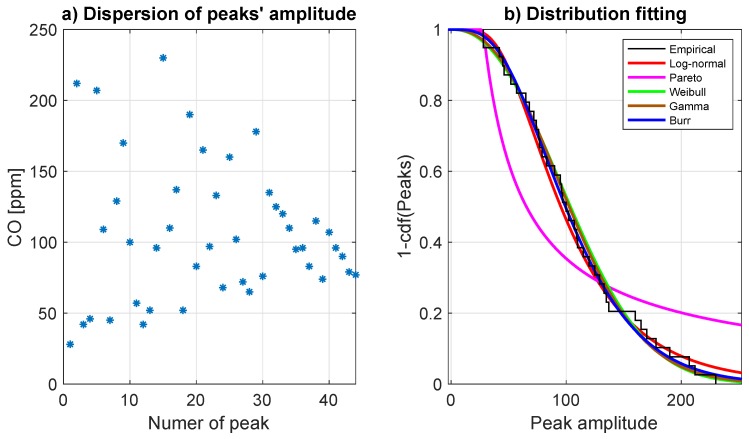
Panel (**a**) shows the dispersion of detected peaks’ amplitudes, whereas panel (**b**) presents the tail of the empirical distribution for peaks’ magnitudes and the theoretical tail of the several fitted distributions.

The dispersion of detected peaks’ amplitudes is plotted in Figure 7a. In order to find the proper class of probability distribution corresponding to the values of the detected peaks, we propose to analyze the behaviour of the empirical tail of the vector presented in Figure 7b. In order to fit the appropriate distribution we have used estimators based on the maximum likelihood method.

In the next step of the analysis we examine the process after the peak (process B, Figure 4). We will check the nature of the process and try to describe it using statistical tools. We would like to highlight that the process of the extinguishing of high CO concentration (process B) takes a much longer time than the process responsible for achieving the maximum concentration (process A).

According to the mining regulation, the blasting procedure should be made at a specific time during the shift. However, there are minor differences between days. We synchronize data according to the maximum value of CO concentration to avoid the “blurring” effect. We experimentally have chosen one hour before the peak and 6.5 h after it as the concentration describing the part of blasting with some excess. The overlapped parts of the CO concentration confirm the pattern existence. While analyzing the carbon monoxide variation data, one can observe the moment during the extinguishing of peaks where the CO concentration reaches the safe 26 ppm level and after some time plateaus.

**Figure 8:**
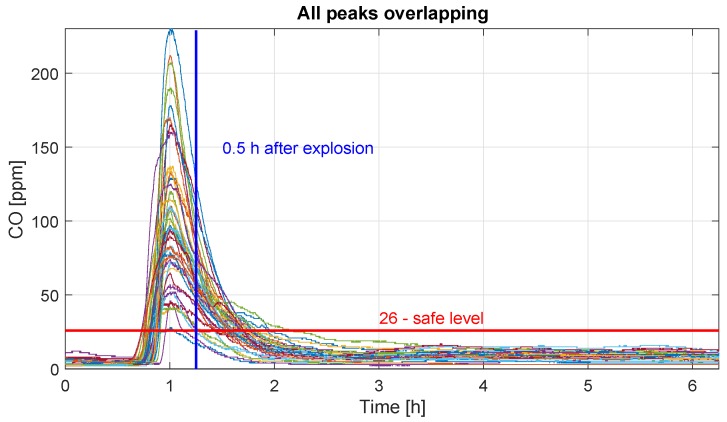
Recorded peaks overlapping according to the fixed time frame. The yellow vertical line indicates 30 min waiting time after blasting and the red horizontal line points the safe level of gas concentration.

**Figure 9:**
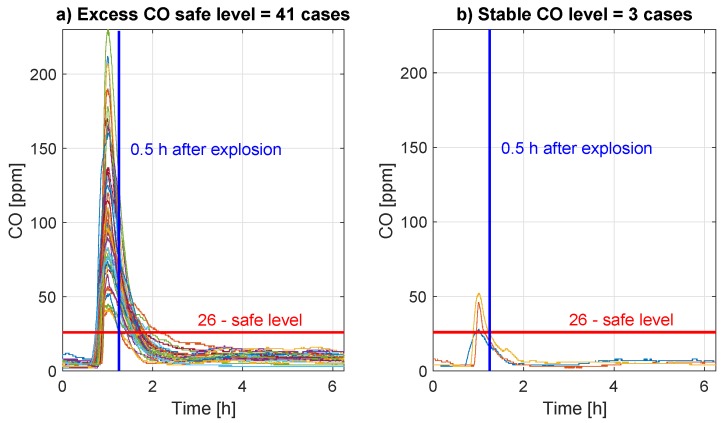
Panel (**a**) shows the processes of CO concentration related to the blasting events in case when the CO concentration does not reach the safe level before 30 min from the blasting moment, whereas panel (**b**) presents the same but in the case when the CO concentration reaches the safe level before 30 min from the blasting moment.

As it was mentioned in Section 1, the time equal to 30 min was the most common waiting time after blasting to allow miners entry into the place. The horizontal red line, see Figure 8 and Figure 9a,b, denotes the limit value of the carbon monoxide concentration allowing a human to stay in such air conditions. The vertical line denotes the time equal to 30 min after the potential explosion. In Figure 9a,b we demonstrate that neither 7% of peaks follow the assumption, that 30 min is good enough to obtain a safe carbon monoxide level; 93% of peaks significantly exceed safety 26 ppm level.

**Figure 10:**
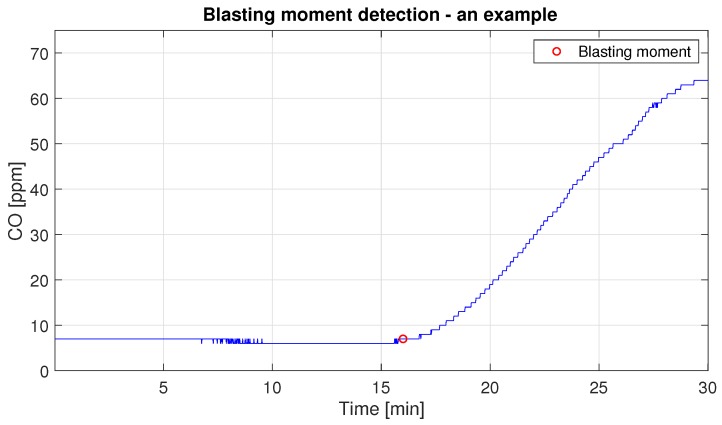
The illustration of the result of the blasting moment location procedure.

In order to properly describe the waiting time for the safe level, we firstly propose to detect the point where the explosion starts. This blasting moment is determined using the behavior of the first derivative of the function. The description of this procedure, as well as the realization with the given data, is presented in Appendix A. In Figure 10 the graphical example of the procedure for the blasting moment detection is presented.

**Figure 11:**
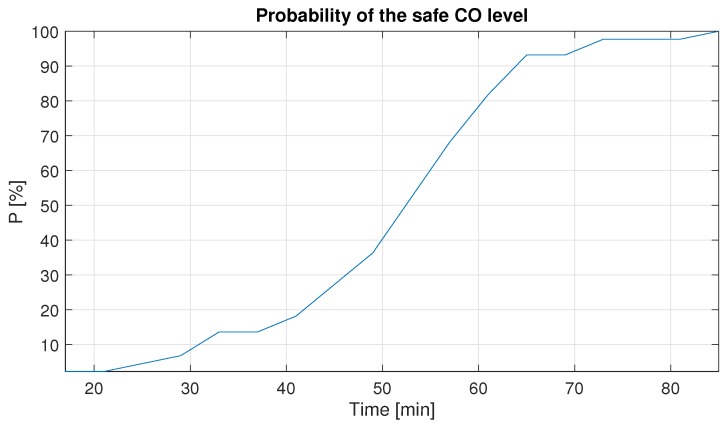
The empirical probability of the time needed to ensure the safe CO level after blasting procedure.

In the next step we take into consideration all peaks and calculate the time when they reach the safe level from the identified blasting moment, indicated in Figure 4. Finally, we select the fraction of the peaks with the carbon monoxide concentration below 26 ppm for a given time. The results of this analysis are presented in Figure 11.

As one can see, the appropriate waiting time during 100% of the peaks, taken from the long-term historical data, to reach the safe 26 ppm level is 85 min. Whereas, the time equal to 65 min gives us 93% assurance that the CO concentration reaches the safe level. After this time, there is a breakdown in the function of probability and for the next 5 min we have no improvement. Thus, the optimal waiting time seems to be 65 min.

**Figure 12:**
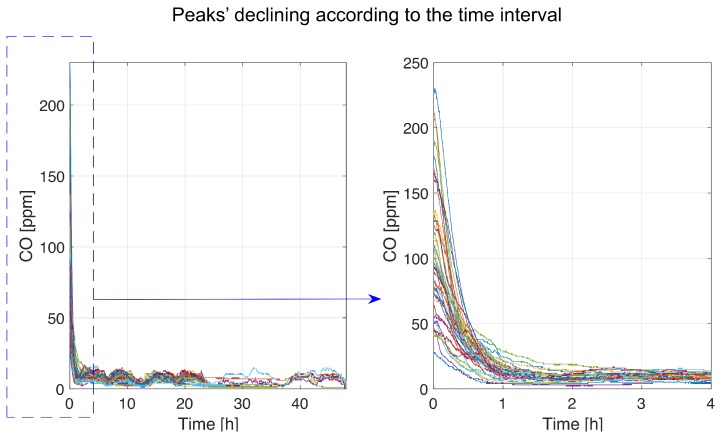
The carbon monoxide concentration variation following maximum values achieved.

As a final step of our analysis, we examine the carbon monoxide concentration data after achieving the maximum value of the peak (process B, Figure 4). This process for all detected jumps is presented in Figure 12.

Similar to the blasting moment identification, there is a need to define the point responsible for the ending of the process B. To obtain the period when the influence of the process B in the total CO concentration can be neglected, we have to find the separation point, matched in Figure 4. It could distinguish the period of high carbon monoxide concentration caused by technological processes and the period when the natural carbon monoxide concentration is more visible. To detect the separation point we use the first local minimum after the peak with the following assumptions:

1. The separation point is not faster than 85 min after the peak, in accordance with the probability (100%) of achieving the sufficient time which brings the safe 26 ppm level.
2. The size of a window responsible for the local minimum is equal to 45 min (chosen arbitrarily).

**Figure 13:**
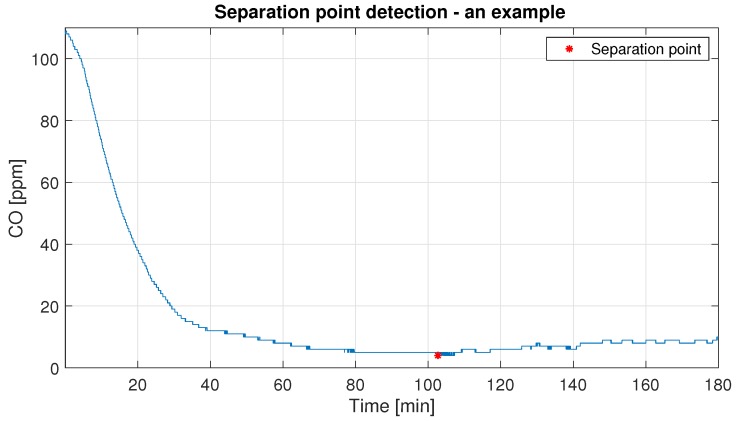
The illustration of the procedure for the separation point location.

In Figure 13 the graphical example of the procedure for the separation point location is presented. Other examples are presented in Appendix B. The median time of "red star" location for the analyzed real-data is 98.46 min after the peak.

Using the location of peak and the location of the separation point, fitting of the extinguishing function has been performed. The exact procedure is described in Section 3.

**Figure 14:**
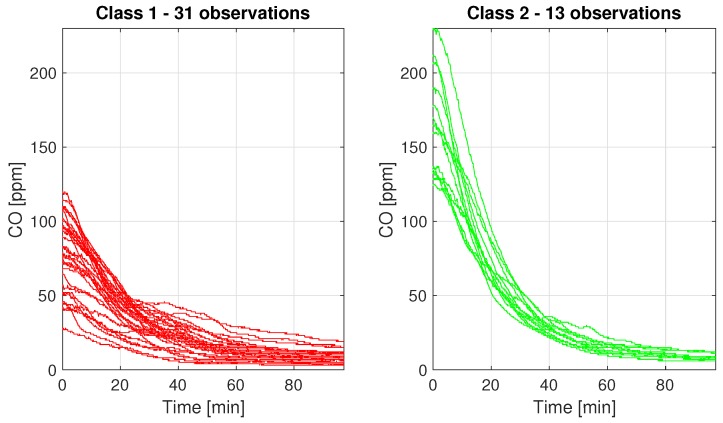
Peaks’ decreasing classification.

In order to demonstrate that the process after the peak can have different behaviors, the classification has been done. The number of classes chosen by the Silhouette algorithm is equal to 2. The K-means clustering algorithm is applied to the matrix of data from the peak location to the separation point. The result of the classification is presented in Figure 14.

**Figure 15:**
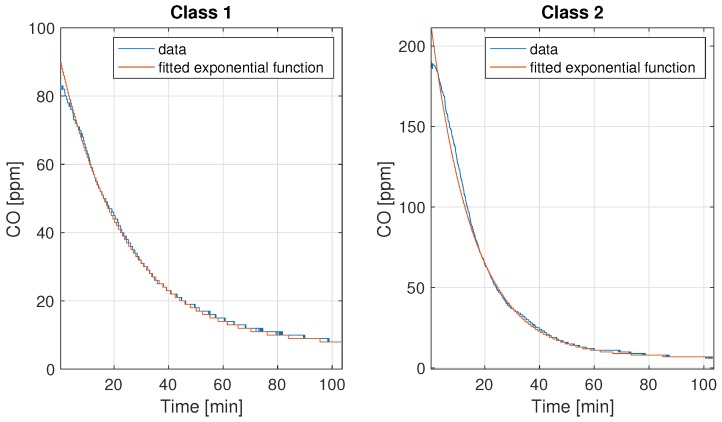
The examples of the exponential function fitting (11)—the process after peak.

In order to describe the nature of the process after the peak, we propose to fit the deterministic function which in the best way describes the data. The best fitting we obtained for the exponential function with different parameters was:

(11) $$\begin{array}{c} f(x)=a\exp(bx)+c, \end{array}$$

where *a* and *b* are scale parameters and *c* is the shift parameter. In Figure 15 we present the representatives of the classes with the fitted exponential function (red colour). Other examples have been presented in Appendix C. For all cases, the determination coefficient ($R^{2}$) of the fitted function was greater than 90%.

**Figure 16:**
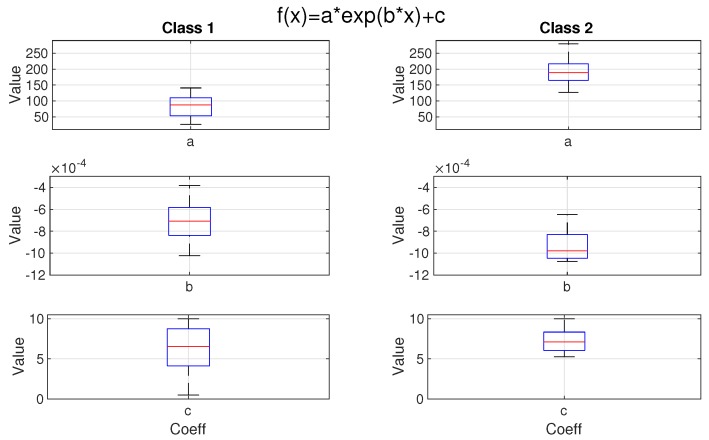
Boxplots of parameters of the fitted exponential functions (11).

The parameters a,b and *c* in Equation (11) are fitted using the least squares method. In Figure 16 we present the boxplots of the estimated parameters for both classes. One can see that these two classes are mainly distinguished by different values of the parameter *a*, which for the second class is much higher. For class 1 the median value of parameter *a* is equal to 87.11, whereas for class 2 the median value of this parameter equal to 189.33. For both classes, we have a similar median value of parameter *c* (for class 1 the parameter c=6.54, while for class 2 parameter c=7.11). However, the distribution of parameter *c* in class 1 is left-skewed, thus we can observe more values which tend to 0.

The value of parameter *a* is closely related to the peak’s amplitude, whereas the value of parameters *c* is equivalent to the level of the plateau observed in the raw data. In practice, we can use this knowledge to precisely predict the time needed to achieve the safe level. To be more precise, after blasting, the CO concentration should be measured and considered as the local peak value (to ensure that it is a peak, the concentration of CO should be measured again and the second measurement should be lower). The obtained peak value gives us the information for which class we should classify it into. Using the definition of the extinguishing function, defined in (11), and the known value of the parameters *a* and *c* (taken from the chosen class), as well as parameter *b*, we can easily predict the process of decreasing. Therefore, based on this information, we can receive a more precise waiting time for a safe level of CO, which is dedicated to this particular situation, in contrast to the statistically calculated 65 min, see Figure 11.

## 5. Discussion and Conclusions

Mining operations in the deep mine, especially in conditions of gas hazards are critically risky; therefore, they are subject to various regulations. Unfortunately, the level of gas hazard and variability of gas phenomena become more and more difficult to handle. Thanks to advanced measurement devices one can measure gas concentration; however, as presented in the paper, concluding from the raw measurement is also very problematic. In the case of CO emission, the daily pattern of CO variation is complicated and the influence coming from blasting is much higher than from other sources, creating the visible peaks in CO concentration. Thus, in this paper, we have focused on this aspect.

According to our best knowledge the long-term real data describing the variation of the carbon monoxide in a deep underground mine have been presented for the very first time. In the paper, the physical explanation of the daily pattern of variability of the CO concentration has been provided and the division for sub-processes (processes A, B and C, see Figure 4) have been proposed. A thorough analysis has been subjected to process B.

The holistic algorithm for detection, extraction and statistical analysis of the key changes in the CO concentration variation (blasting moments, peaks localization, separation points) has been presented. The validation by comparison with seismic data has been proposed. Four of the seismic events were not recognized in CO concentration variation; on the other hand, one of the CO peaks was not registered by the seismic monitoring system. The physical explanations of these divergences have been given.

It has been noticed that daily patterns are different and gas concentration value reaches a safe level in a different way and at a different time.

It has been found that peak amplitudes can be modeled by the Burr distribution. The knowledge about peak amplitude distributions allows for the identification of the expected value of CO concentration caused by the blasting event, that might be helpful during mine operations and ventilation planning.

The probability of achieving a safe value after blasting has been calculated (probability vs. time). It has appeared that after 85 min from the explosion, the gas concentrations for all days have reached the safe value; however, after 30 min the probability of the safe value of CO concentration is 30% only. Nevertheless, the optimal waiting time, which ensures a high probability of safe CO level is 65 min.

The segments related to the CO peaks blanking (process B) have been extracted from the data. By Silhouette criterion and K-means clustering, we have found that there are two clusters of behaviour. Both of them can be described by the exponential function, well imitating the segment behaviours, which parameters a,b and *c* differ depending on the class. The main difference between classes occurs between peaks’ amplitudes and the scale parameter *a* of the exponential function holds this property. It can be used to predict the time needed to achieve a safe CO level after blasting. What is more, the parameter c≈7 ppm represents the CO gas hazard not related to the blasting procedures.

From the practical point of view, the present methodology can be applied for the CO gas analysis in other mining workings where blasting procedure is used, as well as for other gases that vary similarly.

## Figures and Tables

**Table 1:** Basic technical parameters of the gas sensor of the carbon monoxide.

Measuring range	0–1000 ppm
Resolution	1 ppm
Response time	<30 s (T90 norm [26])
Stability	1%
Selectivity ratio	H${}_{2}$S: 6.7%, SO${}_{2}$: 0%, NO: 10%, NO${}_{2}$%: 0, CL${}_{2}$: 0%, H${}_{2}$: 60%,
	HCN: 0%, HCL: 0%, C${}_{2}$H${}_{4}$: 75%
Life cycle	3 years
Working temperature range	−20 °C÷+50 °C
Relative humidity range	0÷95% (without condensation)
Dimensions	180 × 120 × 70 mm
Power supply	12V DC, 10 mA
Feature of explosion-proof construction	I M1 Ex ia I Ma
Weight	1.3 kg
Degree of construction protection	IP65
Transmission standard	RS485
Operation status indication	2 LEDs

**Figure A1:**
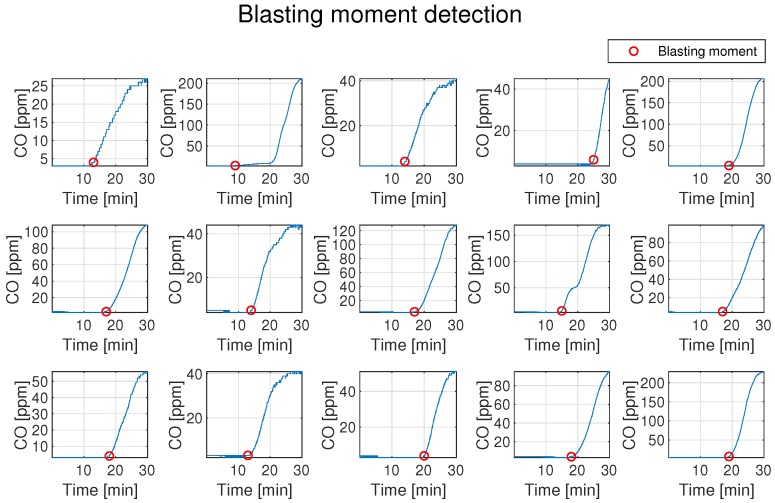
The blasting moment detection—Part I.

**Figure A2:**
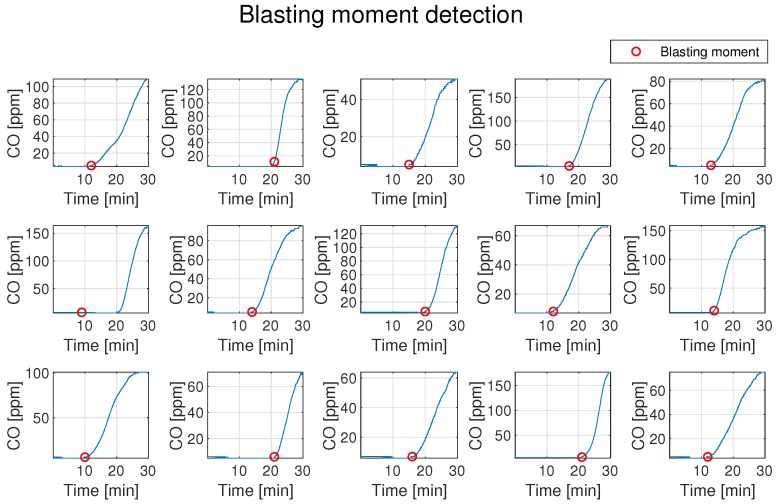
The blasting moment detection—Part II.

**Figure A3:**
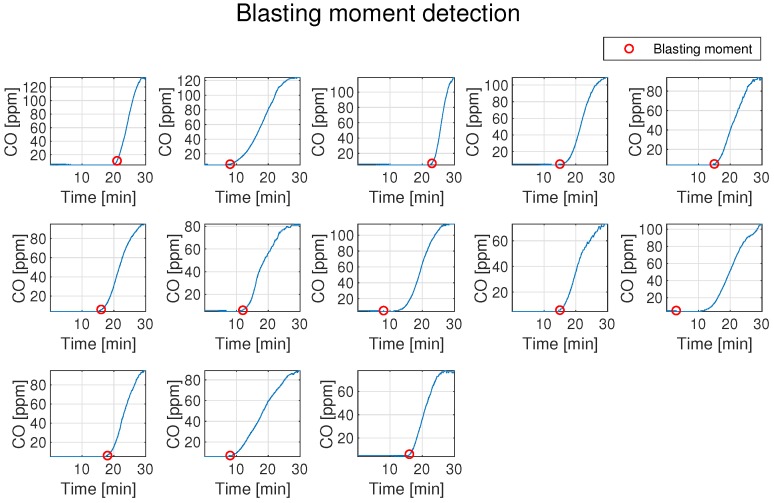
The blasting moment detection—Part III.

**Figure A4:**
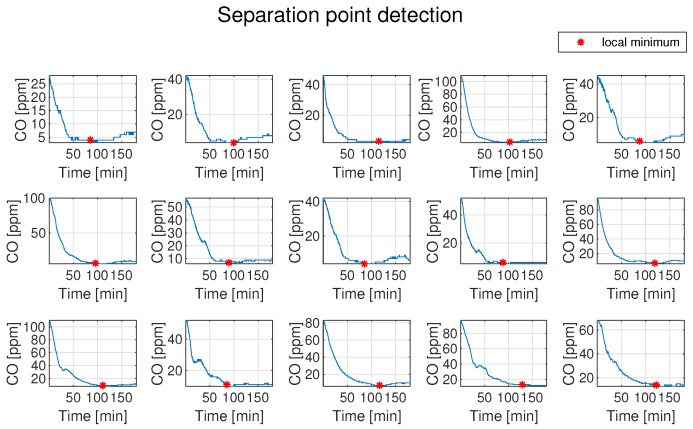
The detection of the separation point—Part I.

**Figure A5:**
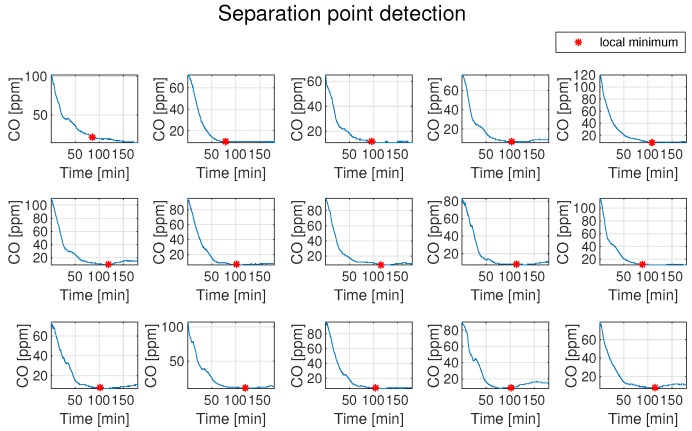
The detection of the separation point—Part II.

**Figure A6:**
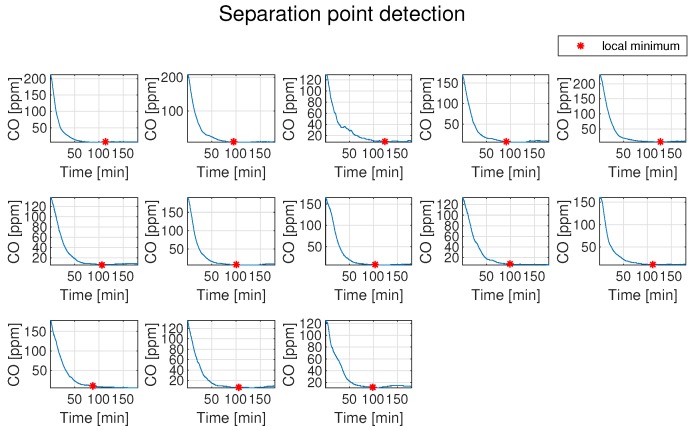
The detection of the separation point—Part III.

**Figure A7:**
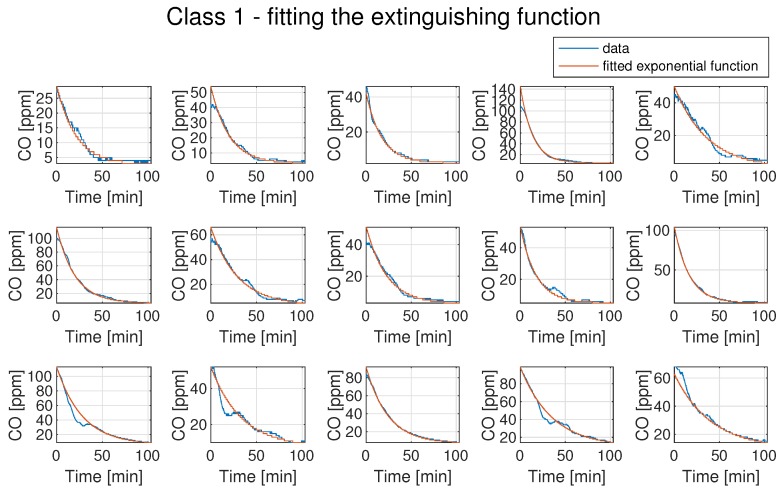
The results of fitting of the deterministic function to the extinguishing process—Part I.

**Figure A8:**
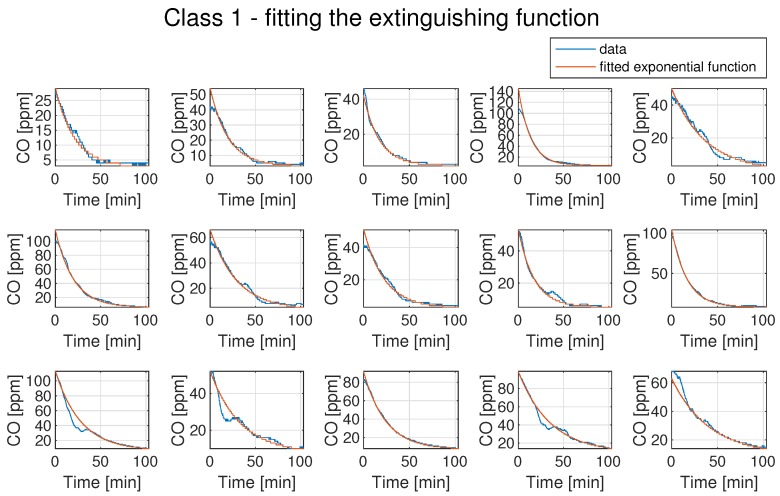
The results of fitting of the deterministic function to the extinguishing process—Part II.

**Figure A9:**
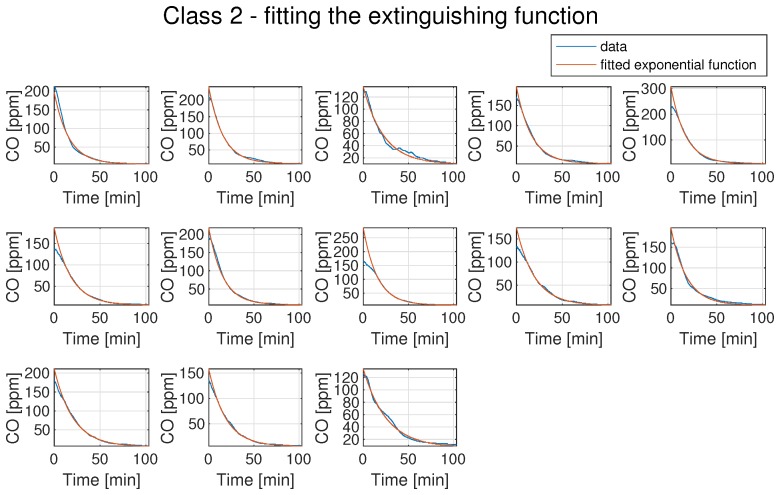
The results of fitting of the deterministic function to the extinguishing process—Part III.

## Appendix A. Blasting Moment Detection

In order to detect the moment of blasting, the property of the monotonic function has been used. We examine the sign of the first derivative on the given window *w*, sized 60 s, namely we calculate $f_{w}^{\prime }\left(x\right)=f\left(x+w\right)-f\left(x\right)$, where *x* is the data sample length 60, according to the size of the moving window. As we know, if the sign of the first derivative is positive, then the function is increasing. In practice we check the sign of the median value of the first derivative in the given non-overlapping window, if it is positive then the beginning of the window we treat as the blasting moment. In Figure A1, Figure A2 and Figure A3 the results of the procedure for real-data have been presented.

## Appendix B. The Separation Point Detection

In order to detect the separation point we use the first local minimum after the peak with the assumptions described in Section 4. The assumptions are as follows:

1. The separation point is minimum 85 min after the peak, in accordance with the probability (100%) of achieving the sufficient time for the safe 26 ppm level.
2. The size of a window responsible for the local minimum is equal to 45 min (chosen arbitrarily).

In Figure A4, Figure A5 and Figure A6 the results of the procedure have been presented.

## Appendix C. Fitting of the Extinguishing Function

In Figure A7, Figure A8 and Figure A9 the results of fitting of the exponential function f(x)=aexp(bx)+c to the analyzed process B have been presented. We present the results for 30 members from class 1 and 13 members from class 2. For all cases, the $R^{2}$ coefficient is greater than 90%, which suggests a good fit of the examined functions. The estimation of the function parameters has been performed using the least squares method.
